# Reply to “comment on: Lessons from the pandemic and the value of a structured system of ultrasonographic findings in the diagnosis of COVID-19 pulmonary manifestations”

**DOI:** 10.31744/einstein_journal/2025CE1697

**Published:** 2025-06-03

**Authors:** Vítor Carminatti Romano, Natália Tavares de Melo Barros Lima, Victor Arantes Jabour, Guilherme Ciconelli Del Guerra, Paulo Rogério Barboza Silvério, Rodrigo Gobbo Garcia, Yoshino Tamaki Sameshima, Miguel José Francisco, Marcos Roberto Gomes de Queiroz

**Affiliations:** 1 Hospital Israelita Albert Einstein São Paulo SP Brazil Hospital Israelita Albert Einstein, São Paulo, SP, Brazil.

Dear Editor:

We thank Dr. Hinpetch Daungsupawong and Dr. Viroj Wiwanitkit for their detailed review of our work: Lessons from the pandemic and the value of a structured system of ultrasonographic findings in the diagnosis of COVID-19 pulmonary manifestations^(1)^ published in einstein. We appreciate your interest in our work, and the time dedicated to sharing your concerns.

We have carefully reviewed your comments, and provide point-by-point responses below.

1. During the SARS-CoV-2 pandemic, fast, dynamic, and non-invasive methods, such as ultrasonography, that can aid in patient triage and diagnostics have become of great importance. Agility on assessment is of critical importance when faced with a large number of patients reaching emergency services throughout the country; hence, ultrasonography was highlighted as an important tool in the fight against COVID-19.
2. In this context, defining objective and reproducible criteria for initial patient and disease assessments would allow physicians to better direct initial treatment and necessary interventions. The versatility of ultrasound, attributed to its availability in both high- and low-resource centers and bedside capabilities, together with its ability to operate within set parameters, makes it a valuable tool for providing objective evidence for disease evaluation.
3. Pulmonary manifestations of the disease, especially peripheral ground-glass opacity, one of the earliest signs of covid infection, are not well observed on chest X-rays. While seen on computed tomography (CT) scans, CT scans are not widely available at most centers and/or cannot be used owing to patient instability. Considering these challenges, ultrasonography has emerged as a valuable diagnostic and patient evaluation tool during the SARS-CoV-2 pandemic.
4. Multiple studies have been conducted to systematize the findings of ultrasonographic examinations of SARS-CoV-2 infections.^2,3^
5. We recognize the necessity for ongoing research with standardized protocols across multiple international centers to produce the best quality of evidence.^(4,5)^
6. A narrative review by Maggi et al.^(5)^ on different lung ultrasound scores in SARS-CoV-2 infection found that ultrasonography is a viable tool for patient triage, disease severity, and prognostic assessment, and helps guide medical decisions. A limitation of ultrasonographic assessment, as reported by the authors, is the existence of multiple scores that lead to a lack of standardization, which should be observed when comparing different scoring methods.
7. We emphasize the importance of the numerous publications on thoracic ultrasonography^(6)^ and integration of appropriate techniques with a thorough understanding of the ultrasonographic manifestations of SARS-CoV-2 infection. This approach ensures that knowledge is preserved and can be effectively applied in clinical practice. By learning from high-quality evidence, we can use thoracic ultrasonography as a scientifically grounded tool with objective, high-quality evidence not only to improve the care of patients with SARS-CoV-2 infection but also to contribute to the management of other pulmonary pathologies.

We confirm that neither the manuscript nor any parts of its content are currently under consideration or published in another journal. All authors have approved the manuscript and agree with its submission to einstein (São Paulo). We have read and understood your journal’s policies, and we believe that neither the manuscript nor the study violates any of these. There are no conflicts of interest to declare.

Thank you for your consideration. We look forward to hearing from you.

Sincerely,
